# Rapid Identification of Dipeptidyl Peptidase-IV (DPP-IV) Inhibitory Peptides from *Ruditapes philippinarum* Hydrolysate

**DOI:** 10.3390/molecules22101714

**Published:** 2017-10-13

**Authors:** Rui Liu, Lei Zhou, Yan Zhang, Nai-Juan Sheng, Zhi-Kang Wang, Ti-Zhi Wu, Xin-Zhi Wang, Hao Wu

**Affiliations:** 1 Jiangsu Key Laboratory of Research and Development in Marine Bio-Resource Pharmaceutics, Nanjing University of Chinese Medicine, Nanjing 210023, China; 18260028797@163.com (N.-J.S.); wzknyt329@163.com (Z.-K.W.); nzywtz24@163.com (T.-Z.W.); wxzatnj@sina.com (X.-Z.W.); 2 College of Pharmacy, Nanjing University of Chinese Medicine, Nanjing 210023, China; 3 Jiangsu Collaborative Innovation Center of Chinese Medicinal Resources Industrialization, National and Local Collaborative Engineering Center of Chinese Medicinal Resources Industrialization and Formulae Innovative Medicine, Nanjing 210023, China; 4 The Affiliated Huai’an of Xuzhou Medical College and The Second People’s Hospital of Huai’an, Huai’an 223002, China; yqzq1980@163.com; 5 Nanjing Normal University, School of Public Administration, Nanjing 210023, China; zhangyan-tcm@163.com

**Keywords:** bioactive peptide, dipeptidyl peptidase-IV, identification, molecular docking, nano-LC-MS/MS, *Ruditapes philippinarum*

## Abstract

Dipeptidyl peptidase-IV (DPP-IV) inhibitory peptides were rapidly identified from *Ruditapes philippinarum* hydrolysate. The hydrolysate was fractionated by ethanol precipitation and preparative reverse phase high-performance liquid chromatography (RP-HPLC). The fraction which showed the highest DPP-IV inhibitory activity was then analyzed by a high-throughput nano-liquid chromatography electrospray ionization tandem mass spectrometry (nano-LC ESI-MS/MS) method, and the sequences of peptides were identified based on the MS/MS spectra against the Mollusca protein data from the UniProt database. In total, 50 peptides were identified. Furthermore, molecular docking was used to identify potential DPP-IV inhibitors from the identified peptides. Docking results suggested that four peptides: FAGDDAPR, LAPSTM, FAGDDAPRA, and FLMESH, could bind pockets of DPP-IV through hydrogen bonds, π-π bonds, and charge interactions. The four peptides were chemically synthesized and tested for DPP-IV inhibitory activity. The results showed that they possessed DPP-IV inhibitory activity with IC_50_ values of 168.72 μM, 140.82 μM, 393.30 μM, and >500 μM, respectively. These results indicate that *R. philippinarum*-derived peptides may have potential as functional food ingredients for the prevention of diabetes.

## 1. Introduction

Diabetes mellitus (DM), a chronic metabolic disease, is a leading public health problem worldwide. Type 2 diabetes mellitus (T2DM) accounts for 90–95% of diabetes cases, and is characterized by insufficient pancreatic insulin secretion and insulin resistance [1]. Dietary patterns have attracted considerable attention and an appropriate diet plays an important role in the treatment of DM. Bio-functional components, such as food-derived peptides, have become a prevalent strategy in the treatment of DM. Food-derived peptides have been reported to possess potential hypoglycemic activity by inhibiting dipeptidyl peptidase-IV (DPP-IV) [2,3,4,5].

Gastric inhibitory polypeptide (GIP) and glucagon-like peptide 1 (GLP-1) have insulinotropic and β-cell-proliferative effects, and are considered to be new strategies in the management of T2DM [6]. DPP-IV is a metabolic enzyme and can quickly cleave GIP and GLP-1, which results in GIP and GLP-1 having a short half-life [7]. Currently, the DPP-IV inhibitory drugs, gliptins (such as sitagliptin, saxagliptin, and alogliptin), are used by type 2 diabetics for the regulation of postprandial serum glucose. Researchers have focused their attention on natural DPP-IV inhibitors, and many naturally-occurring DPP-IV inhibitory peptides from dietary food, such as milk, fish, and rice have been reported [4,8,9,10].

Functional foods are good sources of peptidic DPP-IV inhibitors. *Ruditapes philippinarum* (*R. philippinarum*) is a commercially-cultured marine bivalve found in the mud-sandy coasts of China, especially in Jiangsu province [11]. *R. philippinarum* is considered a typical functional food with both medicinal and nutritional effects. *R. philippinarum* was first documented to have hypoglycemic activity in *Jia You Ben Cao* (*Song* dynasty), and has been used for nearly 1000 years. In the present study, we attempted to identify DPP-IV inhibitory peptides from *R. philippinarum* protein hydrolysates and investigate their sequences and content.

Traditional strategies used for peptide purification and identification are based on assay-directed fractionation [12], which is time-consuming and labor-intensive. Currently, high-resolution LC-MS/MS is a suitable method for the analysis of samples with a complex composition, such as protein hydrolysates, extracts of animal-derived TCMs, and neuropeptides in neurological organs [13,14,15]. Therefore, high-throughput nano-liquid chromatography electrospray ionization tandem mass spectrometry (nano-LC ESI-MS/MS) was used to rapidly resolve peptide components in the complex mixture in the present study. Peptide sequences in the active fraction were identified by searching MS/MS spectra against a protein database. The structure and bio-activity of identified peptides were confirmed by comparing synthetic peptides with the calculated sequences [14]. Molecular docking was then carried out to investigate the potential interactions between the identified peptides and the active site of DPP-IV, and to identify DPP-IV inhibitory peptides from all identified peptides. Finally, the structure and DPP-IV inhibitory activity of screened peptides were confirmed according to their NMR spectrum. The aim of this study was to rapidly explore DPP-IV inhibitory peptides from *R. philippinarum* using LC-MS/MS and molecular docking.

## 2. Results and Discussion

### 2.1. Preparation of Ruditapes Philippinarum Hydrolysates (RPHs) and Determination of DPP-IV Inhibitory Activity

**Figure 1:**
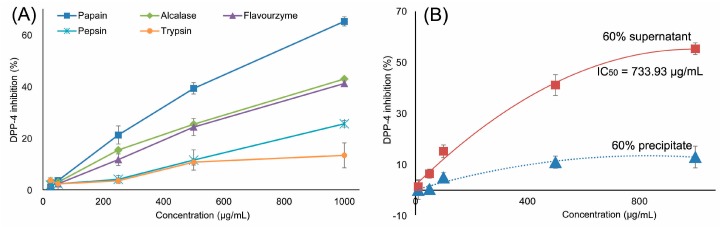
(**A**) DPP-IV inhibitory activities of RPHs produced by different proteases. The IC_50_ of RPHs produced by Papain is 672.12 μg/mL; (**B**) DPP-IV inhibitory activities of 60% precipitate and supernatant, and the IC_50_ of 60% supernatant is 733.93 μg/mL.

Five proteases including papain, trypsin, pepsin, flavourzyme, and alcalase were used to hydrolyze the *R. philippinarum* proteins under optimal conditions. The DPP-IV inhibitory activity of different RPHs was then determined. The RPHs using different proteases exhibited different DPP-IV inhibitory activity (Figure 1). The bioactivity of RPHs followed the order: papain > alcalase > flavourzyme > pepsin > trypsin. The DPP-IV inhibitory activity of peptides was related to the bond cleavage sites of proteins hydrolyzed by proteases. These findings indicated that papain was the most efficient protease to release DPP-IV inhibitory peptides from *R. philippinarum* proteins, and as shown in Figure 1A, papain-generated RPHs displayed the greatest DPP-IV inhibition with an IC_50_ of 672.12 μg/mL. These results were similar to those obtained in an investigation of silver carp protein hydrolysates, in which the papain-generated silver carp protein hydrolysates had the highest DPP-IV inhibitory activity, while trypsin had the lowest [4].

### 2.2. DPP-IV Inhibitory Activity of Fractions

Papain-generated RPHs were precipitated by 60% ethanol (*v*/*v*), and the supernatant fraction showed higher DPP-IV inhibition compared with the precipitate fraction (Figure 1B). This indicated that DPP-IV inhibitory activity may be related to the molecular weight and hydrophobicity of the peptides. Large molecular weight peptides or proteins normally do not dissolve in 60% ethanol (*v*/*v*), and show lower DPP-IV inhibitory activity. Peptides in the supernatant which may contain 2–20 amino acid residues or a molecular weight lower than 2 kDa usually show good bioactivity [15,16,17]. Thus, after discarding the large molecular weight peptides or proteins of RPHs, the short peptides contained in the supernatant inhibited DPP-IV activity.

**Figure 2:**
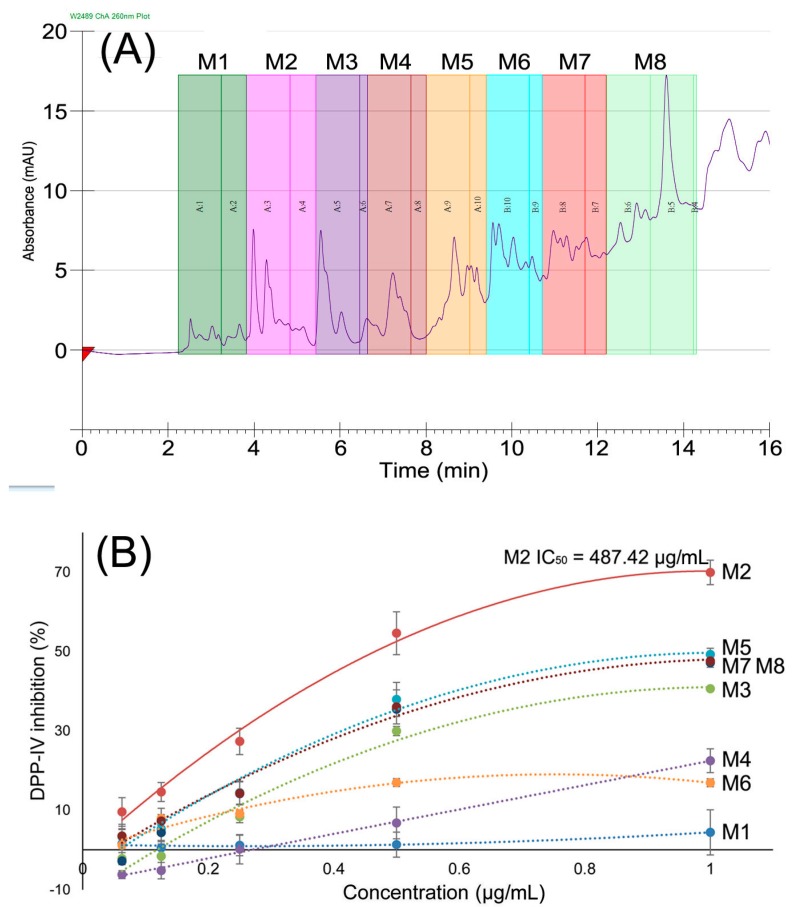
(**A**) RP-HPLC profile of the supernatant of papain-produced RPH; (**B**) DPP-IV inhibitory activities of subfractions from 60% supernatant of RPHs separated by RP-HPLC; M2 showed DPP-IV inhibitory activity of 487.42 μg/mL.

Furthermore, the supernatant fraction was separated by preparative RP-HPLC, and eight fractions were obtained (M1–M8), as shown in Figure 2A. The DPP-IV inhibitory activity of fraction M2 was significantly higher than the other fractions, with an IC50 value of 487.42 μg/mL (Figure 2B). The HPLC profile of the RPH supernatant fraction indicated its complex peptide mixture. According to the classic bioactive peptide investigation strategy, further bioassay, direct isolation or purification was necessary to obtain purified active peptides, and the Edman degradation method and MS/MS were carried out to identify the amino acid sequences. However, this classic investigation strategy is a low through-put method that is both time-consuming and labor-intensive. Therefore, high through-put methods for peptide mixture research are important, and high-resolution nano-LC-MS/MS-based peptide identification from a peptide mixture is a good method for peptide characterization.

### 2.3. Identification of Peptides in M2

Nano-LC-MS/MS was used to identify the peptides in M2. The total ion chromatogram (TIC) was first obtained, then doubly-charged ions were fragmented by collision-induced dissociation (CID) and a series of MS/MS spectra were generated. In total, 50 peptides were identified by de novo sequencing and protein database searching based on the MS/MS spectra. All identified peptides are shown in Table S1. 

**Figure 3:**
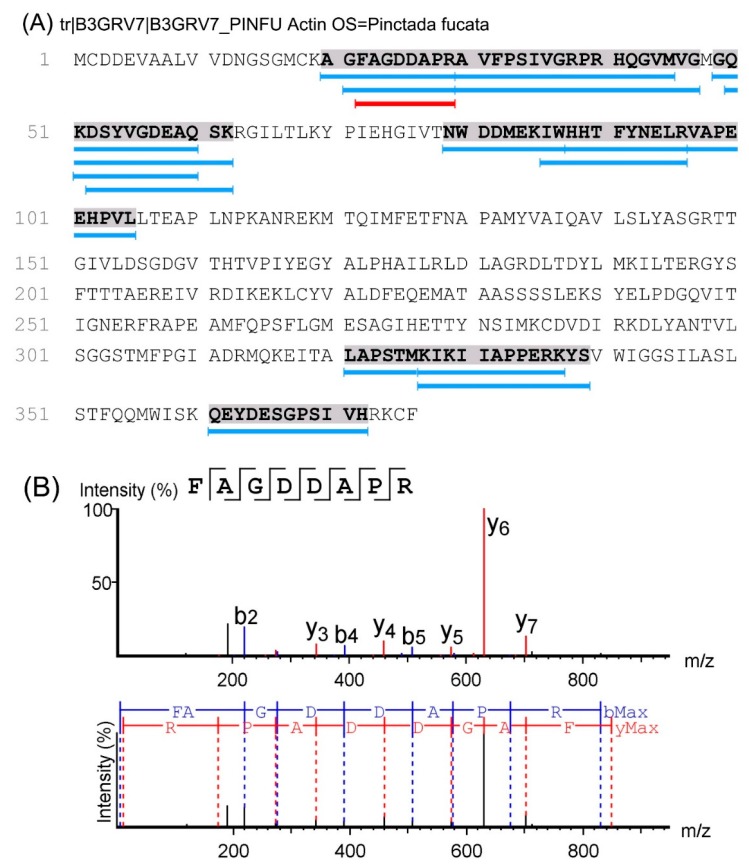
(**A**) Sequence of actin and the distribution of identified peptides. (**B**) Identification of peptide FAGDDAPR (F22–R29).

**Figure 4:**
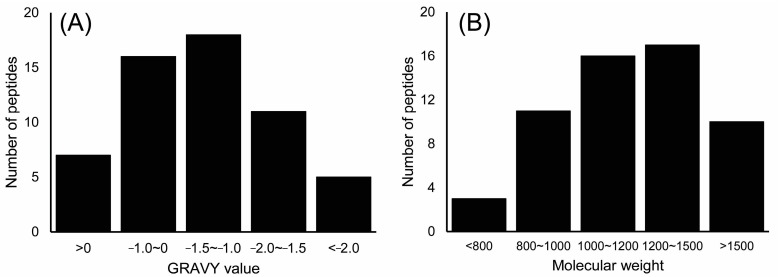
GRAVY index value (**A**) and Mw distribution (**B**) of the identified peptides based on the total number of peptides.

When taking an octopeptide as an example, its primary sequence of FAGDDAPR was identified based on the *m*/*z* 847.38 ion (Figure 3). The sequence of FAGDDAPR was calculated based on the y ion series of *m*/*z* 175.12, *m*/*z* 272.17, *m*/*z* 343.21, *m*/*z* 458.23, *m*/*z* 573.26, *m*/*z* 630.28, and *m*/*z* 701.32, and the b ion series of *m*/*z* 219.11, *m*/*z* 276.13, *m*/*z* 391.16, *m*/*z* 506.19, *m*/*z* 577.22, and *m*/*z* 674.27 in the MS/MS spectra. Other peptides in M2 were deduced from their y and b ion series in their MS/MS spectra. Furthermore, peptide features such as molecular weight (Mw) and the grand average of hydropathicity (GRAVY) index value were evaluated as shown in Figure 4. When considering the global peptides, the Mws of the identified peptides ranged from 618.75 to 2533.30 Da, and in 82.4% of these peptides, the Mw was lower than 1500 Da. This indicated that these peptides may be potentially active due to their lower Mw, which might be easier for crossing the barrier and exerting biological effects. The GRAVY index value was employed to evaluate the hydrophilic and hydrophobic character of the identified peptides. As shown in Figure 4A, 87.7% of the identified peptides were hydrophilic with a GRAVY index value lower than 0.

As shown in Table S1, the 57 identified peptides were derived from actin, myosin, tropomyosin and others. Peptides were dropped from certain areas of the proteins following protease hydrolysis. When taking actin as an example, the identified peptides were dropped mainly from five areas, A_20_-G_47_, G_49_-K_62_, N_79_-L_105_, K_327_-S_339_, and Q_361_-H_372_ of actin (as shown in Figure 3A).

### 2.4. Molecular Docking

**Figure 5:**
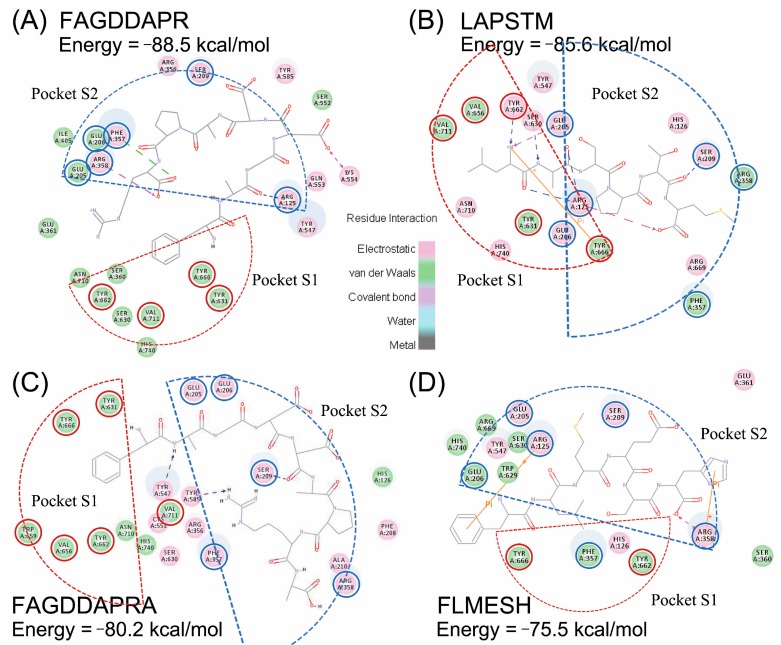
The binding model of peptide inhibitors to DPP-IV in docking studies. Best docking position of peptides FAGDDAPR (**A**); LAPSTM (**B**); FAGDDAPRA (**C**); and FLMESH (**D**) at the DPP-IV pocket. Red circles represent the amino acid residues belong to Pocket S1, and blue circles represent the amino acid residues belong to Pocket S2.

Currently, molecular docking is extensively used for virtual investigation of the interactions between ligands and receptors, which can help to reveal the binding modes and the residues involved in binding interactions. In order to identify potential DPP-IV inhibitors, binding models for interactions between the active sites of DPP-IV, and the inhibitors have been proposed in several investigations [18]. In the present study, a molecular docking strategy was used to screen the 57 identified peptides and identify DPP-IV inhibitory peptides. As a result, four peptides, FAGDDAPR, LAPSTM, FAGDDAPRA, and FLMESH, with potential DPP-IV inhibitory activity were obtained, which could “fit” into the pockets of DPP-IV. The interactions between these four DPP-IV inhibitory peptides and DPP-IV were studied by CDOCKER in Discovery Studio 4.0 (Accelrys Software Inc., San Diego, CA, USA), and the CDOCKER energies were also determined. As shown in Figure 5, the pink circles represent residues involved in hydrogen-bond, charge or polar interactions, and the green circles represent residues involved in van der Waals interactions. DPP-IV comprises a hydrophobic S1 pocket (Tyr631, Val656, Trp659, Tyr662, Tyr666, Val711) and a charged S2 pocket (Arg125, Glu205, Glu206, Phe357, Ser209, Arg358) [19]. Peptides occupy the S1 and S2 pocket and interact with amino acid residues. Octapeptide FAGDDAPR showed the lowest CDOCKER energy with −88.5 kcal/mol, and had the highest DPP-IV inhibitory activity compared with the other three peptides. FAGDDAPR formed interactions with Arg125, Arg358, and Phe357 in the S2 pocket. The CDOCKER energy of hexapeptide LAPSTM was −85.6 kcal/mol. LAPSTM formed 5 hydrogen-bonds with Ser630, Arg125, Ser209, and Try662. Ser630 is the active site of the enzyme, Try662 is a binding site in S1 pocket, and both Arg125 and Ser209 are the binding sites in S2 pocket. The amino-group of Leu in LAPSTM also formed a π-π interaction with Tyr666. Therefore, LAPSTM may exert its DPP-IV inhibitory activity through the above-mentioned interactions. The CDOCKER energy of nonapeptide FAGDDAPRA was −80.2 kcal/mol. FAGDDAPRA bound Ser209 in the S2 pocket, and also formed hydrogen-bond interactions with Tyr547 and Tyr585. Hexapeptide FLMESH did not form any hydrogen-bonds with DPP-IV, but two π-π interactions with Arg125 and Arg358 in pocket S2, and showed the highest CDOCKER energy of −75.5 kcal/mol among the four peptides. Other amino acid residues involved in hydrogen bond, charge, or polar interactions, or van der Waals interactions are represented by pink circles and green circles in Figure 5B.

According to the CDOCKER energy, the inhibitory activity order was as follows: FAGDDAPR > LAPSTM > FAGDDAPRA > FLMESH. However, DPP-IV inhibitory activity determination of the synthetic peptides showed that LAPSTM had the highest DPP-IV inhibitory activity, which may be related to the binding between LAPSTM and DPP-IV pocket sites.

NMR spectra and data were shown in Supplementary Data S1–S5, which could confirm the amino acid sequences of four peptides.

### 2.5. DPP-IV Inhibitory Activity Determination of Synthesized Peptides

**Figure 6:**
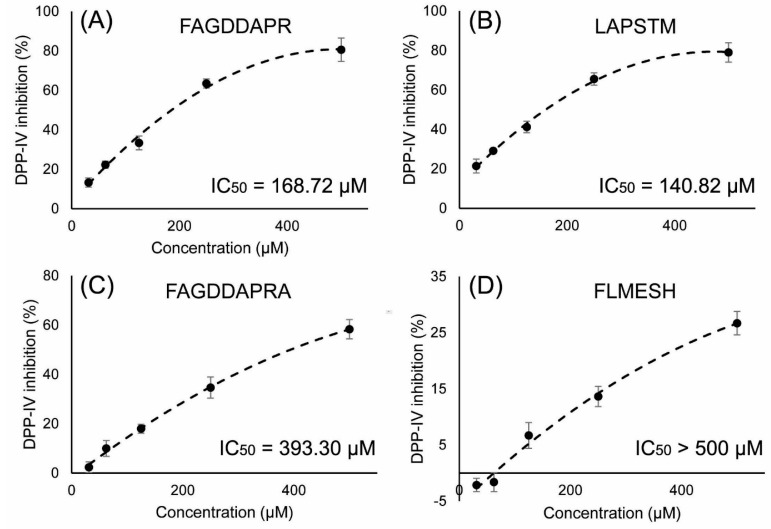
DPP-IV inhibitory activity of the four peptides: (**A**) FAGDDAPR; (**B**) LAPSTM; (**C**) FAGDDAPRA; and (**D**) FLMESH.

FAGDDAPR, LAPSTM, and FAGDDAPRA were hydrolyzed from actin, and FLMESH was identified by de novo sequencing. The four peptides were synthesized with purity >98% and with the appropriate molecular weight (Figures S1 and S2). The DPP-IV inhibitory activity of these chemically—synthesized peptides was determined as described in Section 2.3. As shown in Figure 6 LAPSTM showed the most potent DPP-IV inhibitory activity with an IC_50_ value of 140.82 μM, followed by FAGDDAPR with an IC_50_ of 168.72 μM, and FAGDDAPRA with an IC_50_ of 393.30 μM. The IC_50_ of FLMESH was >500 μM.

**Figure 7:**
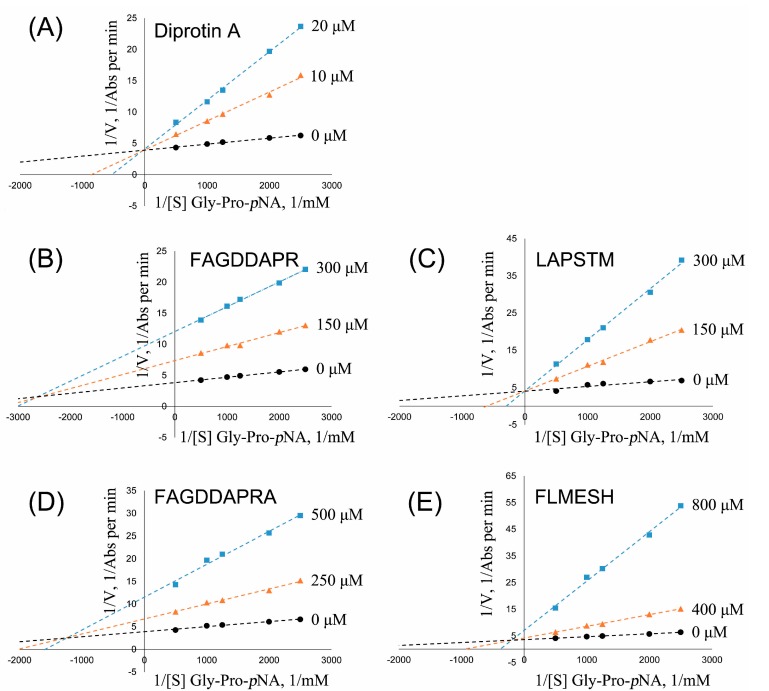
Lineweaver—Burk double-reciprocal plot for DPP-IV activity in the absence and presence of four peptides at different inhibitory concentrations. (**A**) Diprotin A (0, 10, and 20 μM) as reference; (**B**) peptide FAGDDAPR (0, 150, and 300 μM); (**C**) peptide LAPSTM (0, 150, and 300 μM); (**D**) peptide FAGDDAPRA (0, 250, and 500 μM); and (**E**) peptide FLMESH (0, 400, and 800 μM).

In addition, the synthesized peptides showed good DPP-IV inhibitory activity were further investigated to determine the modes of action on the enzyme, as shown in Figure 7. Positive control diprotin A showed IC_50_ of 13.10 μM and behaved as a competitive inhibitor (Figure 7A), which was reported in the literature [4]. FAGDDAPR, FAGDDAPRA, and FLMESH (Figure 7B,D,E) displayed a competitive/non-competitive mixed-type inhibition mode for DPP-IV enzyme. It was indicated that the three peptides exhibited their DPP-IV inhibitory activities by both binding to the active site of DPP-IV and outside the catalytic center. LAPSTM (Figure 7C) displayed a competitive-type inhibition mode for DPP-IV enzyme, which indicated that LAPSTM exhibited its inhibitory activity by binding to the active site of DPP-IV.

Hydrophobic amino acids at the N-terminal of peptides are thought to increase the DPP-IV substrate specificity [20]. Thus, Leu and Phe in the N-terminal position of the four peptides may enhance their specificity for DPP-IV. In addition, the presence of an Ala residue at the penultimate N-terminal position is one of the structural features associated with DPP-IV inhibitory activity [7]. As shown in Figure 5, the carbonyl of Ala in LAPSTM and FAGDDAPR binds Arg125 of DPP-IV through hydrogen-bonds, and the amino of Ala in FAGDDAPRA binds Tyr547 through hydrogen-bonds. In addition, only one amino acid residue different from FAGDDAPR, nonapeptide FAGDDAPRA, is ended with Ala in the C-terminal, which leads to a totally different binding site in fitting DPP-IV in the S2 pocket (Figure 5). C-terminal Ala may affect the binding mode of FAGDDAPRA, which appears to have a negative effect on DPP-IV inhibitory potency. In another study by Zhang, one more Ala residue at the N-terminal position, compared with LAPSTM in the present study, demonstrated that heptapeptide ALAPSTM derived from silver carp actin showed a weak DPP-IV inhibitory effect [4]. It can be speculated that Ala in the N-terminal may occupy the S2 pocket and prevent hydrogen-bond formation between Leu-Ala and DPP-IV. According to the binding interaction between LAPSTM and DPP-IV, it is suggested that Leu-Ala at the N-terminal position of the peptide can easily access the pocket of DPP-IV and form various bonds with DPP-IV, as a dipeptide Leu-Ala has been reported to possess DPP-IV inhibitory activity with an IC_50_ value of 91 μM [21]. Therefore, Leu and Phe at the first position, and Ala at the second position, may have played an important role in exerting the DPP-IV inhibitory activity of peptides in the present study.

## 3. Materials and Methods

### 3.1. Materials

*R. philippinarum* was collected from the Lüsi aquaculture regions of Jiangsu province, China in June 2016, and was examined by Prof. Xihe Wan (Institute of Oceanology and Marine Fisheries, Nantong, China) prior to processing.

Papain (from papaya, 8 × 10^5^ units/g of protein), trypsin (from porcine pancreas, 2.5 × 10^5^ units/g of protein), pepsin (from porcine gastric mucosa, 1 × 10^4^ units/g of protein), flavourzyme (from guanyloribonuclease, 2 × 10^4^ units/g of protein), and alcalase (from *Bacillus licheniformis*, 2 × 10^5^ units/g of protein) were purchased from YIFEIXUE Biological Tech., Ltd. (Nanjing, China). Gly-Pro-*p*-nitroanilide, diprotin A (Ile-Pro-Ile), and porcine DPP-IV (>10 units/mg protein) were purchased from Sigma Chemical Co. (St. Louis, MO, USA). Mass spectrometry (MS)-grade water, acetonitrile (ACN), and trifluoroacetic acid (TFA) were purchased from Tedia Company Inc. (Fairfield, CT, USA). All other reagents were obtained from Sigma. All reagents were of analytical grade.

### 3.2. Preparation of Hydrolysates

The preparation of hydrolysates was carried out as previously described with minor modifications [4]. *R. philippinarum* flesh was collected and stored at −20 °C until processing. The flesh was cut into small pieces and extracted twice with water for 1 h each time to remove polysaccharides and other water-soluble components. The residues were then homogenized with distilled water (5% *w*/*w*). The homogenates were pre-incubated at the appropriate temperature for each proteinase hydrolysis. All reactions were incubated for 4 h under optimal conditions according to the manufacturer’s protocol for each enzyme: papain, pH 7.0 and 55 °C at an enzyme/substrate ratio of 1% (*w*/*w*), trypsin, pH 8.5 and 50 °C at an enzyme/substrate ratio of 1% (*w*/*w*), pepsin, pH 2.0 and 37 °C at an enzyme/substrate ratio of 1% (*w*/*w*), flavourzyme, pH 7.0 and 50 °C at an enzyme/substrate ratio of 10% (*w*/*w*), alcalase, pH 8.5 and 50 °C at an enzyme/substrate ratio of 1% (*w*/*w*), and neutrase, pH 7.0 and 45 °C at an enzyme/substrate ratio of 6% (*w*/*w*). After incubation, the hydrolysates were heated to 100 °C for 10 min to deactivate the enzyme, and then centrifuged. The supernatants were adjusted to pH 8.0, lyophilized, and stored at −20 °C for further use.

### 3.3. DPP-IV Inhibitory Activity Assay

DPP-IV inhibitory activity was measured as previously described by Zhang [4] with minor modifications. Briefly, a 25 μL sample (diluted in 100 mM Tris-HCl buffer at pH 8.0) was mixed with 25 μL Gly-Pro-*p*-nitroanilide (1.6 mM) and pre-incubated at 37 °C for 10 min. Subsequently, 50 μL of DPP-IV (8 U/L) was added and the mixture was incubated at 37 °C for 1 h. The reaction was terminated by adding 100 μL of 1 M sodium acetate buffer at pH 4.0. The absorbance of each reaction was measured at 405 nm. Diprotin A (Ile-Pro-Ile, IPI) was used as a reference inhibitor. The percent DPP-IV inhibition was calculated according to the following equation:

$$\mathrm{DPP-IV inhibition}\left(\%\right)=\left[1-\frac{A_{\mathrm{sample}}}{A_{\mathrm{blank}}}\right]\times 100\%$$

### 3.4. R. philippinarum Hydrolysate Fractionation

Firstly, pure ethanol was added to the *R. philippinarum* hydrolysates (RPHs) to achieve a concentration of 60% (*v*/*v*), and left at 4 °C for 12 h. The mixture was then centrifuged, precipitated and the supernatant was collected and freeze-dried for further use.

The fraction displaying the highest DPP-IV inhibitory activity following ethanol fractionation was further separated by RP-HPLC using a Waters 2545-2489 HPLC system (Waters Corp., Milford, MA, USA), and a Waters Xbridge Prep C_18_ (5 μm, 19 × 150 mm) was used for separation. Elution was carried out with 2% B for 3 min, a linear gradient from 2 to 35% B for another 9 min, and kept at 35% B for 4 min, with a flow rate of 10 mL/min (eluting solvent A: 0.1% TFA in pure water; B: 0.1% TFA in methanol). The detection wavelength was set at 220 nm for all compounds. The HPLC profile is shown in Figure 2A. In total, eight fractions (M1–M8) were collected by the Waters Fraction Collector III (Waters Corp.).

### 3.5. Peptide Characterization by Nano-LC-MS/MS

The subfraction with the highest DPP-IV inhibitory activity following HPLC separation was analyzed using a Dionex 3000 nano-LC system tandem LTQ-Orbitrap Velos Pro (Thermo Fisher Scientific, Waltham, MA, USA) with high energy collision dissociation (HCD). Lyophilized M2 was re-dissolved in acetonitrile/formic acid/water (2/0.2/98, *v*/*v*/*v*), and 5 μL M2 was loaded onto a self-packed 5 μm Reprosil C_18_AQ column (75 μm × 150 mm). The mobile phase consisted of acetonitrile/formic acid/water (2/0.2/98, *v*/*v*/*v*) for buffer A and acetonitrile/formic acid/water (80/0.2/20, *v*/*v*/*v*) for buffer B. Processed samples were analyzed using a 150 min gradient from 2% to 30% of B. The LTQ-Orbitrap was operated in data-dependent acquisition mode to automatically alternate between a full scan (*m*/*z* 300–2000) in the Orbitrap and HCD MS/MS scans in the linear ion trap. Helium was used as the collision gas for HCD. The normalized collision energy was 35% and the activation time was 30 ms. Unless otherwise stated, three replicate measurements were obtained at each MS setting. Data acquisition was controlled by Xcalibur 2.0.7 and Tune 2.4 software (Thermo Fisher Scientific, Bremen, Germany).

Peptide identification was carried out with PEAKS Studio (Version 8.0, Bioinformatics Solutions Inc., Waterloo, ON, Canada). MS/MS spectra were used to search the Mollusca_uniprot-taxonomy_6447 database (downloaded on 20 March 2017). The specified enzyme was chosen as the non-enzyme and up to two missed cleavages were allowed. The false discovery rate (FDR), average local confidence (ALC) and MS/MS tolerance were set at 1, 90% and 0.5 Da, respectively. Oxidation of methionine (+15.9949) and acetylation of the protein N-terminus (+42.0106) were specified as variable modifications.

### 3.6. Molecular Docking

DPP-IV in the present study was retrieved from RCSB PDB (entry code: 1WCY) with the diprotin A inhibitor. The binding site for 1WCY was experimentally verified and the coordinates of the validated diprotin A complex with 1WCY are available online [22]. The bound diprotin A inhibitor from the 1WCY structure was removed for docking experiments. The default parameter values were similar to the “Prepare Protein” protocol from Discovery Studio 4.0 for DPP-IV model preparation.

Molecular docking studies were carried out using the crystal structure of the human DPP-IV complex with diprotin A, and were performed using CDOCKER with Discovery Studio 4.0. To perform flexible docking, all torsion angles of the peptides were set to be free. The DPP-IV binding site was defined as a sphere encompassing protein residues within 10 Å of the original ligand. Docking was performed with 30 conformations of each peptide and rated by the placement method of Proxy Triangle and the rescoring function of London dG. The best conformations were used to calculate the CDOCKER energy value.

### 3.7. Peptide Synthesis and DPP-IV Inhibitory Activity Determination

In order to identify the four DPP-IV inhibitory peptides, peptides were purified from M2 by RP-HPLC using a Waters 2545-2489 HPLC system. And then four peptides were characterized by NMR.

The DPP-IV inhibitory peptides identified by nano-LC MS/MS and NMR were synthesized using the solid phase method and purified with HPLC by the GenScript Corporation (Nanjing, China). The DPP-IV inhibitory activity of these chemically synthesized peptides was determined as described in Section 3.3.

### 3.8. Statistical Analysis

The data presented are the mean ± S.D. of three independent experiments.

## 4. Conclusions

In the present study, it was demonstrated, for the first time, that peptides in the active fraction were identified by high-throughput nano-LC-MS/MS, and DPP-IV inhibitory peptides were determined by molecular docking. The obtained peptides were then synthesized and their DPP-IV inhibitory activity was determined. Of the peptides identified, the hexapeptide LAPSTM showed the highest DPP-IV inhibitory activity with an IC_50_ of 140.82 μM. Furthermore, the interactions between the active peptides and DPP-IV were also investigated by molecular docking. Docking simulation suggested that all four DPP-IV inhibitory peptides form interactions with Arg125, and LAPSTM forms five hydrogen bonds with Ser630, Arg125, Ser209, and Try662. Ser630 is the active site of the enzyme, and the other three peptides can also bind DPP-IV through interactions. Even though the activity of the peptides identified in this study was only moderate, it may be conceived that they could possibly be used as starting points for structural modifications to increase the DPP-IV inhibitory activity in the future. The method developed in this study may potentially be applied to rapidly explore functional peptides from seafood proteins.

## Supplementary Materials

The supplementary materials are available online.

## References

[1] Verspohl E.J. (2012). Novel pharmacological approaches to the treatment of type 2 diabetes. Pharmacol. Rev..

[2] Nongonierma A.B., FitzGerald R.J. (2014). Susceptibility of milk protein-derived peptides to dipeptidyl peptidase IV (DPP-IV) hydrolysis. Food Chem..

[3] Nongonierma A.B., Paolella S., Mudgil P., Maqsood S., FitzGerald R.J. (2017). Dipeptidyl peptidase IV (DPP-IV) inhibitory properties of camel milk protein hydrolysates generated with trypsin. J. Funct. Foods.

[4] Zhang Y., Chen R., Chen X.L., Zeng Z., Ma H.Q., Chen S.W. (2016). Dipeptidyl peptidase IV-inhibitory peptides derived from Silver Carp (*Hypophthalmichthys molitrix* Val.) proteins. J. Agric. Food Chem..

[5] Neves A.C., Harnedy P.A., O’Keeffe M.B., FitzGerald R.J. (2017). Bioactive peptides from Atlantic salmon (*Salmo salar*) with angiotensin converting enzyme and dipeptidyl peptidase IV inhibitory, and antioxidant activities. Food Chem..

[6] Mentlein R. (2005). Therapeutic assessment of glucagon-like peptide-1 agonists compared with dipeptidyl peptidase IV inhibitors as potential antidiabetic drugs. Expert Opin. Investig. Drugs.

[7] Harnedy P.A., O’Keeffe M.B., FitzGerald R.J. (2015). Purification and identification of dipeptidyl peptidase (DPP) IV inhibitory peptides from the macroalga *Palmaria palmata*. Food Chem..

[8] Lacroix I.M.E., Li-Chan E.C.Y. (2014). Isolation and characterization of peptides with dipeptidyl peptidase-IV inhibitory activity from pepsin-treated bovine whey proteins. Peptides.

[9] Lacroix I.M.E., Li-Chan E.C.Y. (2012). Dipeptidyl peptidase-IV inhibitory activity of dairy protein hydrolysates. Int. Dairy J..

[10] Hatanaka T., Inoue Y., Arima J., Kumagai Y., Usuki H., Kawakami K., Kimura M., Mukaihara T. (2012). Production of dipeptidyl peptidase IV inhibitory peptides from defatted rice bran. Food Chem..

[11] Liu R., Wu H., Cheng J.M., Wang X.Z., Yang X.L., Qiu Y.Y., Wang L.C. (2015). The status and prospect of comprehensive utilization of bivalve derived from Jiangsu coastal area. J. Nanjing Univ. TCM.

[12] Robinson S.D., Norton R.S. (2014). Conotoxin gene superfamilies. Mar. Drugs.

[13] Buchberger A., Yu Q., Li L. (2015). Advances in mass spectrometric tools for probing neuropeptides. Annu. Rev. Anal. Chem..

[14] Liu R., Huang Q., Duan J.A., Zhu Z.H., Liu P., Bian Y., Tao J.H., Qian D.W. (2017). Peptidome characterization of the antipyretic fraction of Bubali Cornu aqueous extract by nano liquid chromatography with orbitrap mass spectrometry detection. J. Sep. Sci..

[15] Liu R., Wang M., Duan J.A., Guo J.M., Tang Y.P. (2010). Purification and identification of three novel antioxidant peptides from *Cornu Bubali* (water buffalo horn). Peptides.

[16] Taniguchi M., Kameda M., Namae T., Ochiai A., Saitoh E., Tanaka T. (2017). Identification and characterization of multifunctional cationic peptides derived from peptic hydrolysates of rice bran protein. J. Funct. Foods.

[17] Ortiz-Martinez M., Gonzalez de Mejia E., García-Lara S., Aguilar O., Lopez-Castillo L.M., Otero-Pappatheodorou J.T. (2017). Antiproliferative effect of peptide fractions isolated from a quality protein maize, a white hybrid maize, and their derived peptides on hepatocarcinoma human HepG2 cells. J. Funct. Foods.

[18] Meduru H., Wang Y.T., Tsai J.J., Chen Y.C. (2016). Finding a potential dipeptidyl peptidase-4 (DPP-4) inhibitor for type-2 diabetes treatment based on molecular docking, pharmacophore generation, and molecular dynamics simulation. Int. J. Mol. Sci..

[19] Juillerat-Jeanneret L. (2014). Dipeptidyl peptidase IV and its inhibitors: Therapeutics for type 2 diabetes and what else?. J. Med. Chem..

[20] Power O., Nongonierma A.B., Jakeman P., FitzGerald R.J. (2014). Food protein hydrolysates as a source of dipeptidyl peptidase IV inhibitory peptides for the manage. Proc. Nutr. Soc..

[21] Lan V.T., Ito K., Ohno M., Motoyama T., Ito S., Kawarasaki Y. (2015). Analyzing a dipeptide library to identify human dipeptidyl peptidase IV inhibitor. Food Chem..

[22] Nongonierma A.B., Mooney C., Shields D.C., FitzGerald R.J. (2014). In silico approaches to predict the potential of milk protein-derived peptides as dipeptidyl peptidase IV (DPP-IV) inhibitors. Peptides.

